# Fluoride resistance capacity in mammalian cells involves complex global gene expression changes

**DOI:** 10.1002/2211-5463.12236

**Published:** 2017-06-05

**Authors:** Shujun Ran, Ning Sun, Yun Liu, Wu Zhang, Yiming Li, Limin Wei, Jia Wang, Bin Liu

**Affiliations:** ^1^ Department of Endodontics and Operative DentistryNinth People's HospitalShanghai Jiao Tong University School of MedicineShanghai Key Laboratory of StomatologyShanghaiChina; ^2^ Department of Physiology and PathophysiologyFudan UniversityShanghaiChina; ^3^ The Ministry of Education Key Laboratory of Metabolism and Molecular MedicineDepartment of Biochemistry and Molecular BiologySchool of Basic Medical SciencesFudan UniversityShanghaiChina; ^4^ Center for Dental ResearchLoma Linda University School of DentistryCAUSA; ^5^ School and Hospital of StomatologyWenzhou Medical UniversityChina

**Keywords:** fibroblasts, fluoride, fluoride resistance, transcriptome

## Abstract

Fluorine is a bone‐seeking element ubiquitously present in the environment and widely used in many oral hygiene products. In humans, excessive intake of fluoride may cause dental and skeletal fluorosis. However, endemic fluorosis does not appear to develop in a proportion of individuals exposed to the same levels of fluoride. The mechanisms by which mammalian cells resist fluoride are still unclear. In this study, we developed strains of mouse L‐929 cells resistant to different levels of fluoride. High‐throughput RNA‐sequencing analyses of the fluoride‐resistant L‐929 cells indicated that massive changes in global gene expression occurred, compared with the wild‐type L‐929 cells. The main biological processes and functions changed were associated with the extracellular region and matrix, response to stress, receptor binding, and signal transduction. This indicated that high doses of fluoride not only exerted stress on L‐929 cells but also induced functional pathways that helped them adapt to the presence of fluoride or to expel it. These data should prove useful in identifying cellular processes or transporters/channels that play central roles in adaptation to or expulsion of fluoride in humans.

**Abbreviations**
ECM: extracellular matrix
FoxO: forkhead box O
FR: fluoride‐resistant
KEGG: Kyoto encyclopedia of genes and genomes
MAPK: mitogen‐activated protein kinase
MTT: methyl thiazolyl tetrazolium
NF‐kappa B: nuclear factor kappa B
OD: optical densities
PI3K: phosphoinositide 3‐kinase
WT: wild‐type

Fluorine is a bone‐seeking element and is ubiquitous in the environment. Compared with chloride and iodide, two halides that have been well studied for their effects on living organisms, much less is known about the biological importance of fluoride and the mechanisms by which cells respond to this anion 1, 2, 3. Since 1950s, fluoride has been successfully used in drinking water as a public health measure for preventing dental caries 4. In addition, fluoride is widely used in many oral hygiene products. For example, most current toothpastes available directly to the consumers contain up to 1450 p.p.m. (76.3 mm) fluoride. On the other hand, accumulated data suggest that fluoride is toxic to bacteria, fungi, plants, animals, and humans at high concentrations or doses 5, 6, 7. The mechanisms by which fluoride is toxic to these different species have been investigated extensively but are yet to be fully understood. In human, excessive intake of fluoride may cause dental and skeletal fluorosis, two most common endemic fluorosis associated with excessive fluoride exposure. Dental fluorosis leads to pitting, perforation, and chipping of the teeth, whereas skeletal fluorosis causes more severe consequences like pains in joints followed by stiffness, which ultimately leads to paralysis 8. Interestingly, endemic fluorosis does not appear to develop in a proportion of individuals exposed to the same levels of fluoride, which suggests that some individuals are less sensitive to and/or have gained a capability to resist fluoride toxicity 9.

Because of its ubiquitous presence in the environment and its toxic effects, organisms most likely have evolved mechanisms of resistance to fluoride. Previous studies suggested that a carrier protein could have already existed within the cellular membrane acting as a barrier to fluoride or as a transporter of fluoride, protecting the activity of intracellular enzymes sensitive to fluoride 10, 11. Membrane transport proteins of the chloride channels, which could be such carrier candidates, are used by many organisms for a range of biological tasks that require movement of Cl^−^ or other inorganic anions across cell membranes 2, 12, 13. Recently, a riboswitch‐controlled subtype of chloride channels referred to as ClC^F^ is identified to function as fluoride‐specific F^−^/H^+^ antiporters in bacteria and lower eukaryotes 14, 15, 16, 17. However, to date, no specific fluoride channels have yet been identified in mammalian cells. The mechanisms that mammalian cells resist fluoride are still unclear.

In the present study, we developed strains of mouse L‐929 cells resistant to different levels of fluoride. High‐throughput RNA‐sequencing analyses of the fluoride‐resistant (FR) L‐929 cells indicated that, compared with the wild‐type (WT) L‐929 cells, massive changes in global gene expressions occurred within the induced FR L‐929 cells. The main biological processes and functions changed were associated with extracellular region and matrix, response to stress, receptor binding, and signal transductions etc., indicating high dose fluoride not only exerted stress on L‐929 cells but also induced functional pathways that helped them adapt to or expel the fluoride. Our data may be valuable in identifying cellular processes and transporters/channels that play central roles in adapting to/expelling fluoride in mammalian cells in the future.

## Materials and methods

### Medium preparation

The culture medium was Eagle's Minimum Essential Medium (MEM; Sigma, St. Louis, MO, USA) supplemented with 10% FBS (Gibco, Grand Island, NY, USA), 100 U·mL^−1^ penicillin, and 100 μg·mL^−1^ streptomycin. The 2000 p.p.m. (105.2 mm) fluoride stock solution was prepared by solving 2.2 g sodium fluoride (Sigma) powders in 500 mL MilliQ water (EMD Millipore, Billerica, MA, USA). Different concentrations of fluoride media (work solution) were prepared by mixing different volume of fluoride stock solution with the culture medium.

### Development of fluoride‐resistant strains of L‐929 cells

Wild‐type L‐929 cells (ATCC CCL‐1, Manassas, VA, USA) were maintained in culture media at 37 °C under a 5% CO_2_ atmosphere. During subculture, cells were treated with 0.25% (wt/vol) trypsin/0.02% (wt/vol) EDTA and then were resuspended and seeded with culture media. Countess Automated Cell Counter (Invitrogen, Grand Island, NY, USA) were used to count cells and measure average cell size.

Fluoride‐resistant L‐929 cells, which are capable of proliferating in media of fluoride concentrations that would otherwise be cytotoxic to WT L‐929 cells, were induced *in vitro* by sequential exposure of WT L‐929 cells to 10 p.p.m. (0.526 mm) fluoride concentration gradient ascending media. The induction was initiated by subcultivating L‐929 cells in fluoride medium of low fluoride concentration. Cell proliferation rate was determined by the methyl thiazolyl tetrazolium (MTT) assay. The induction cycles continued till the proliferation rate of the WT L‐929 cells in the fluoride media became normal and were repeated at a higher fluoride concentration till the desired FR L‐929 cell strain was established.

### cDNA library preparation and RNA sequencing

Poly(A) mRNA was isolated using beads containing oligo (dT) from total RNA. Purified mRNA was then fragmented and used as templates. The first‐strand cDNA was synthesized with random hexamer primers and the second‐strand cDNA was synthesized using buffer, dNTPs, RNase H, and DNA polymerase I. Short double‐stranded cDNA fragments were purified with a QIAquick PCR purification kit (Qiagen, Hilden, Germany) and ligated to Illumina sequencing adaptors. The amplified library was sequenced on an Illumina HiSeq™ 2000 (Illumina, San Diego, CA, USA) sequencing machine by paired‐end sequencing.

### Differentially expressed gene analysis

Differentially expressed genes (fold change ≥ 2) between WT and 30 p.p.m. FR L‐929 cells were identified using the significance of digital gene expression profiles.

### Quantitative real‐time RT‐PCR

Total RNA was extracted using the Trizol reagent (Life Technologies, Grand Island, NY, USA) according to the manufacturer's instructions. RNA was reverse‐transcribed into cDNAs by the AMV Reverse Transcription System (Promega Corporation, Madison, WI, USA). Quantitative RT‐RCR was performed with the SYBR Green QPCR system (Roche Applied Science, Mannheim, Germany) with GAPDH as an internal control.

### Statistical analysis

Experimental data were reported as mean ± SEM. To compare between the different of two groups, two‐tailed Student's *t*‐test was used. When *P* value is less than 0.05, significant differences were determined.

## Results

### Effect of fluoride on growth of WT L‐929 cells

**Figure 1:**
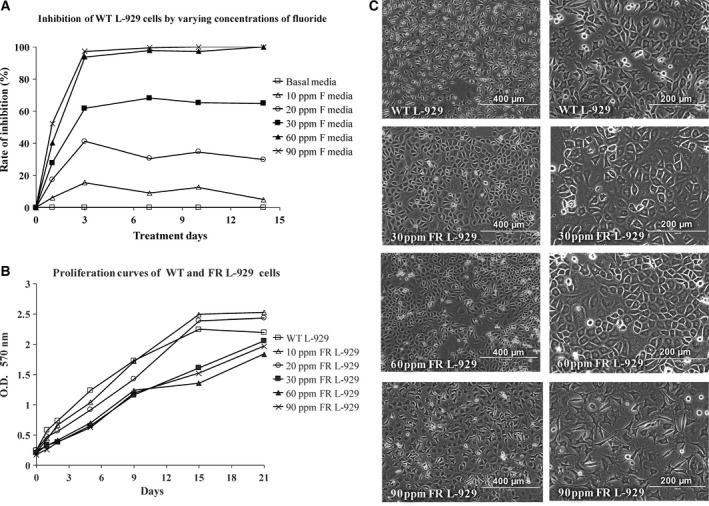
Inducement of FR strains of L‐929 cells. (A) Inhibition rate of WT L‐929 cells in varying concentrations of fluoride media. WT L929 cells were cultured in media with various concentrations of fluoride. MTT assay was performed according to scheduled date and the OD at the wavelength of 570 nm were obtained by a microplate reader (Bio‐Rad Benchmark, Hercules, CA, USA). (B) Proliferation curves of WT and induced FR strains of L‐929 cells. WT L‐929 cells were cultured in fluoride‐free media while FR strains were cultivated in fluoride media. MTT test was performed according to scheduled date and the 570 nm OD were documented. (C) WT and induced FR strains of L‐929 cells.

Wild‐type L‐929 cells were shocked with different concentrations of fluoride by exposing cells to prepared fluoride media. MTT assay was performed according to scheduled time duration and the optical densities (OD) at the wavelength of 570 nm were obtained. As shown in Fig. 1A, different concentrations of fluoride showed divergent inhibition effects on the growth of WT L‐929 cells. Compared with fluoride‐free media, 10 and 20 p.p.m. (0.526 and 1.052 mm) fluoride caused slight to moderate reduction in cell growth. Cells tended to adapt to present fluoride concentration around 3 days after the treatment. Inhibition was pronounced with 30 p.p.m. (1.579 mm) fluoride and showed no obvious debilitation during 2‐week observation period. In the presence of 60 p.p.m. (3.158 mm) or higher concentrations of fluoride, extended cell death occurred quickly after the addition of fluoride and almost all cells died within 3 days.

### Development of FR L‐929 cells

Initially the WT L‐929 strain was exposed to 5 p.p.m. (0.263 mm) fluoride media till cell proliferation rate was back to normal, which we considered that the strain was adapted to 5 p.p.m. (0.263 mm) fluoride. Next, cells were subcultured in media with 10 p.p.m. (0.526 mm) fluoride. Four weeks later, the growth rate was normal again and 10 p.p.m. FR strain was obtained. Such cells were then stepwise exposed to 10 p.p.m. (0.526 mm) gradient ascending fluoride media from 20 p.p.m. (1.052 mm) to 90 p.p.m. (4.737 mm). Fluoride sensitivity was determined by MTT test when cell confluence reached 90% to confirm the adaptation of cells to fluoride. Cells would not be exposed to higher concentration of fluoride until the growth rate became normal and the adaptation was obtained. The proliferation curves of WT and FR L‐929 cells were showed in Fig. 1B.

As shown in Fig. 1C, there were no gross morphological discrepancies observed between the WT and FR strains of L‐929 cells except for the cell size. WT strain appeared slimmer than the FR strains. The average cell diameters of the WT strain, 30, 60, and 90 p.p.m. FR strains were about 12.4, 14.9, 16.6, and 15.9 μm, respectively.

### High‐throughput RNA‐sequencing analyses of global gene expression changes in the 30 p.p.m. FR L‐929 cells

**Figure 2:**
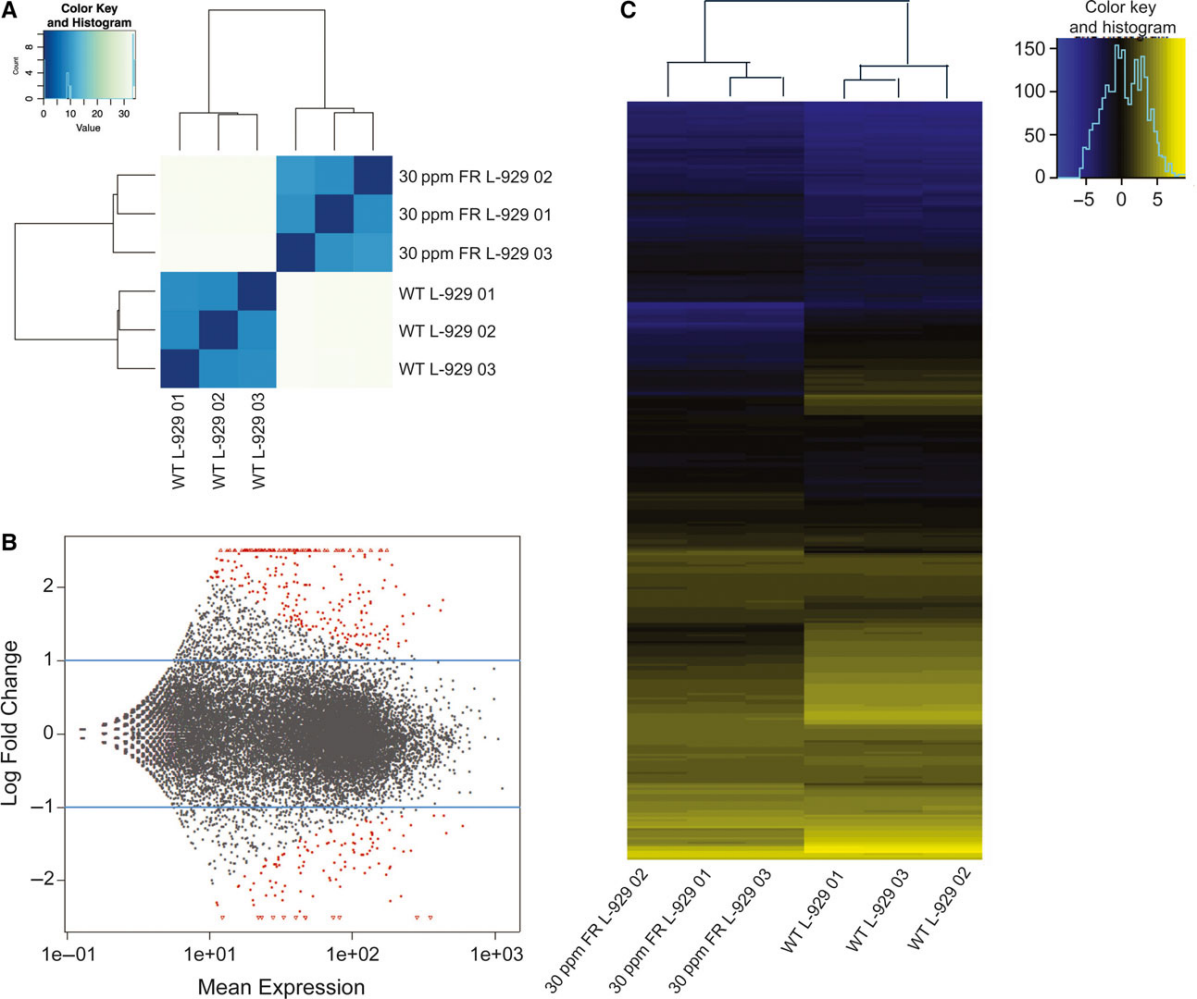
Analyses of global gene expression pattern of the fluoride resistance L‐929 cells by RNA‐Seq. (A) Global gene expression correlation study of the WT and 30 p.p.m. FR L‐929 cells. (B) Fold changes of gene expression levels between the WT and 30 p.p.m. FR L‐929 cells. Each dot represents expression levels of a single coding gene. (C) Heat map of the genes with greater than 2‐fold changes in 30 p.p.m. FR L‐929 cells compared to those of the WT L‐929 cells.

To investigate the major mechanisms or pathways that mediate fluoride resistance of the FR L‐929 cells, we examined the global gene expression changes in the 30 p.p.m. FR L‐929 strains versus WT L‐929 cells. The reason we chose the 30 p.p.m. FR strains for RNA‐seq analysis was that the fluoride concentration is right in between the starting 10 p.p.m. (0.526 mm) and the highest 90 p.p.m. (4.737 mm) fluoride concentration so that the gene expression changes may be significant but not biased. We used three batches of independently cultured WT and 30 p.p.m. FR L‐929 cells in the study. Cluster analysis of the global gene expression patterns showed that the three independent 30 p.p.m. FR L‐929 cells naturally grouped together, indicating the 30 p.p.m. (1.579 mm) fluoride exerted significant changes on gene expressions within L‐929 cells (Fig. 2A). Compared with WT L‐929 cells, there are total 385 genes that exhibited at least twofold changes in 30 p.p.m. FR L‐929 cells, of which 265 genes were up‐regulated and 120 genes were down‐regulated in expression level (Fig. 2B, C and Table S1).

### Gene ontology analyses of RNA‐seq results for the 30 p.p.m. FR L‐929 cells

**Figure 3:**
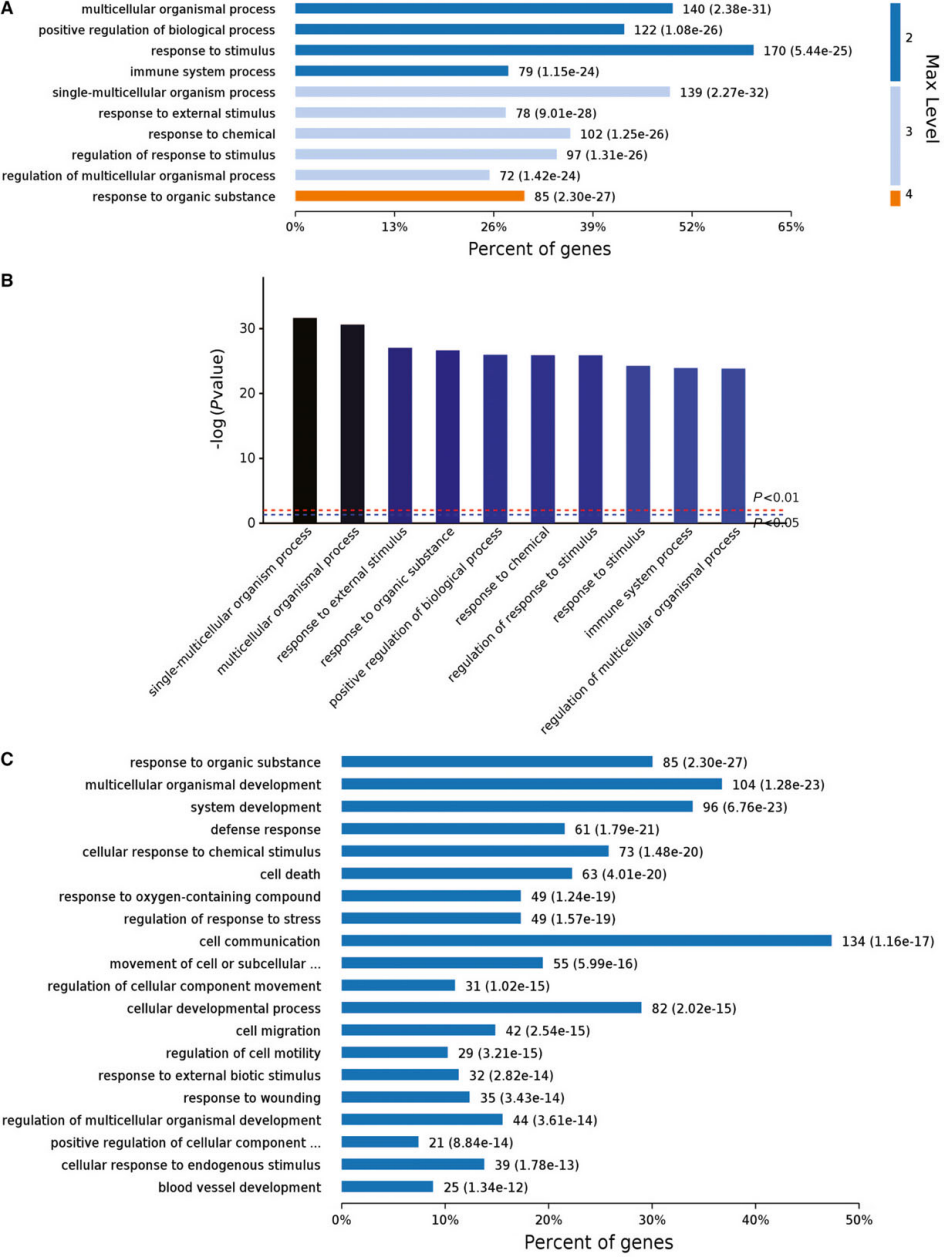
Biological process analyses of the RNA‐seq results. (A) Major biological processes the 385 changed genes were involved. (B) Ranking of top 10 biological processes by *P* values. (C) Deeper level 4 analyses of the biological processes the 385 changed genes were involved.

We next performed gene ontology analyses of the 385 genes whose expressions were significantly changed in the 30 p.p.m. FR L‐929 cells. Analyses of the biological processes that the 385 genes were involved showed that many genes possibly belong to stress response (Fig. 3A). Of the 10 most significant biological processes (with the highest *P* values), 6 of them were related to responses to external stimuli (Fig. 3B), further supporting the above conclusion. These results indicated that 30 p.p.m. (1.579 mm) fluoride was first a strong external stress for the survival of the L‐929 cells. Level 4 analyses of the biological process showed that the top three biological processes (with the highest percent of genes) were ‘cell communication’, ‘multicellular organismal development’, and ‘system development’ (Fig. 3C). This result was somewhat ‘interesting’ and may suggest that the L‐929 cells tried communicated with each other and act as a whole system to resist the external 30 p.p.m. (1.579 mm) fluoride stress.

**Figure 4:**
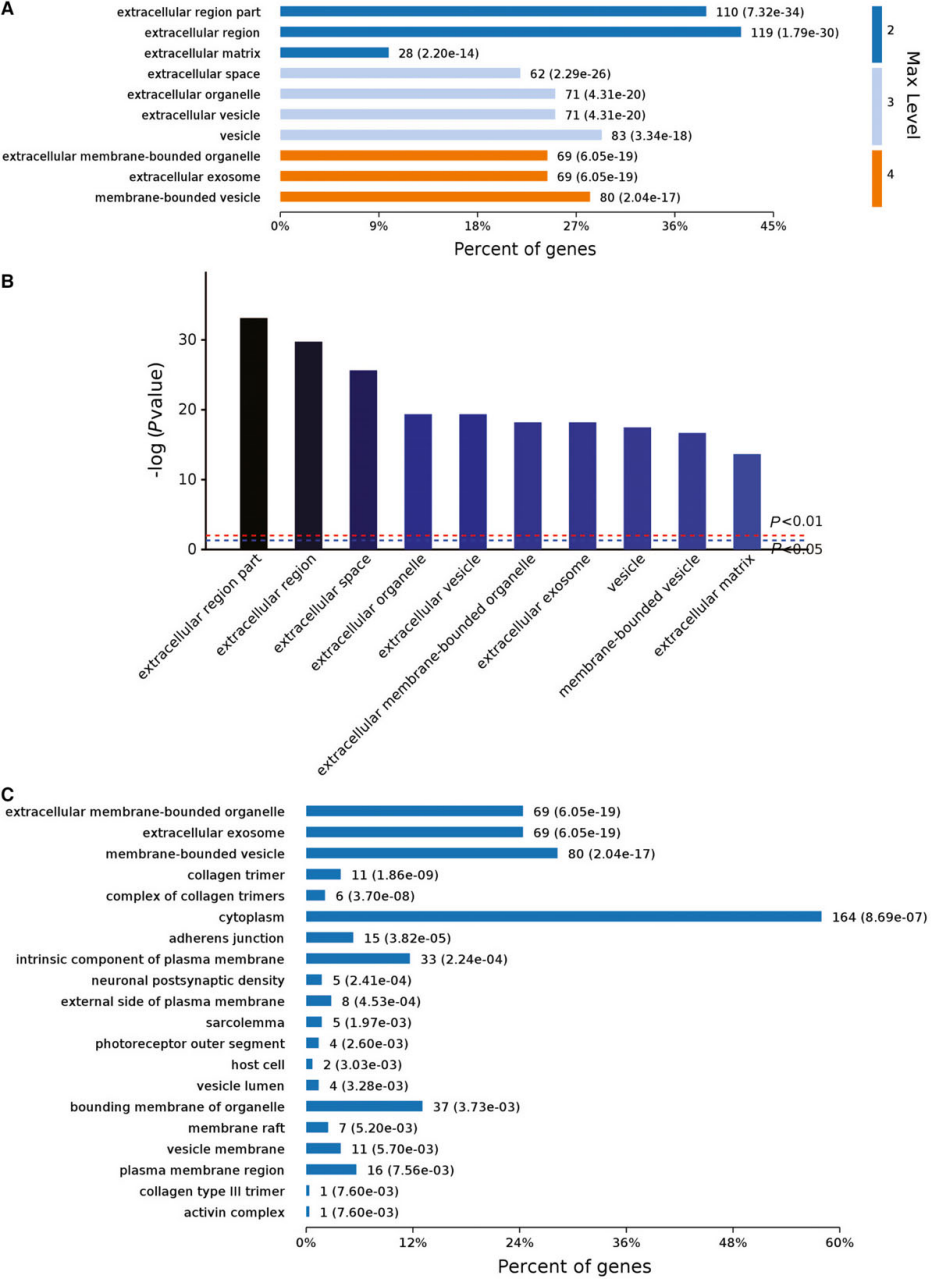
Cellular component analyses of the RNA‐seq results. (A) Major cellular components the 385 changed genes belong to. (B) Ranking of top 10 cellular components by *P* values. (C) Deeper level 4 analyses of the cellular component the 385 changed genes belong to.

Analyses of the cellular components that the 385 genes belong to showed that most of them were closely associated with the extracellular compartment, including extracellular matrix (ECM), organelles, and vesicles etc. (Fig. 4A,B). Deeper level 4 analyses of the cellular components indicated that there were 164 genes which also belong to ‘cytoplasm’ (Fig. 4C). These results showed that the 30 p.p.m. (1.579 mm) fluoride stress elicited massive rearrangements of extracellular molecules as well as their cytoplasmic counterparts. The above information suggested that the L‐929 cells made a lot of changes across the cellular membrane to adapt the toxic stress of the 30 p.p.m. (1.579 mm) fluoride.

**Figure 5:**
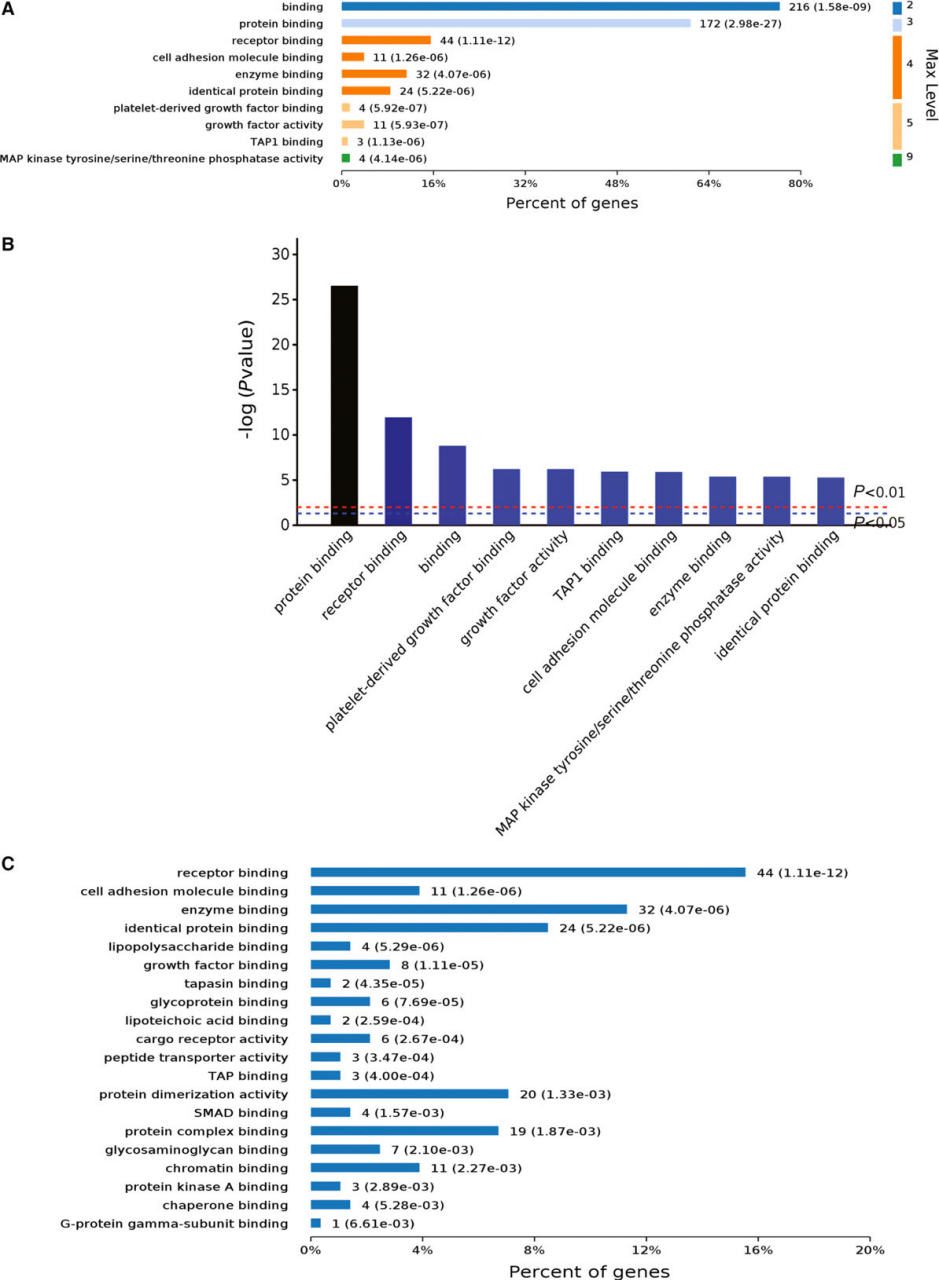
Molecular function analyses of the RNA‐seq results. (A) Major molecular functions the 385 changed genes were involved. (B) Ranking of top 10 molecular functions by *P* values. (C) Deeper level 4 analyses of the molecular functions the 385 changed genes were involved.

Analyses of the molecular functions the 385 genes were associated with showed that a majority of them were involved in protein and receptor binding (Fig. 5A,B). Deeper level 4 analyses of the molecular functions further showed that these genes belong to a list of the protein‐binding activities, including receptor, cell adhesion molecules, lipopolysaccharides, glycoprotein, and enzyme binding etc. (Fig. 5C). These results suggested that the 30 p.p.m. (1.579 mm) fluoride elicited many signaling transduction activities in the L‐929 cells, which could also play very important roles in helping the cells in adaptation to the toxic fluoride rich environment.

### KEGG pathway analyses of RNA‐seq results for the 30 p.p.m. FR L‐929 cells

**Figure 6:**
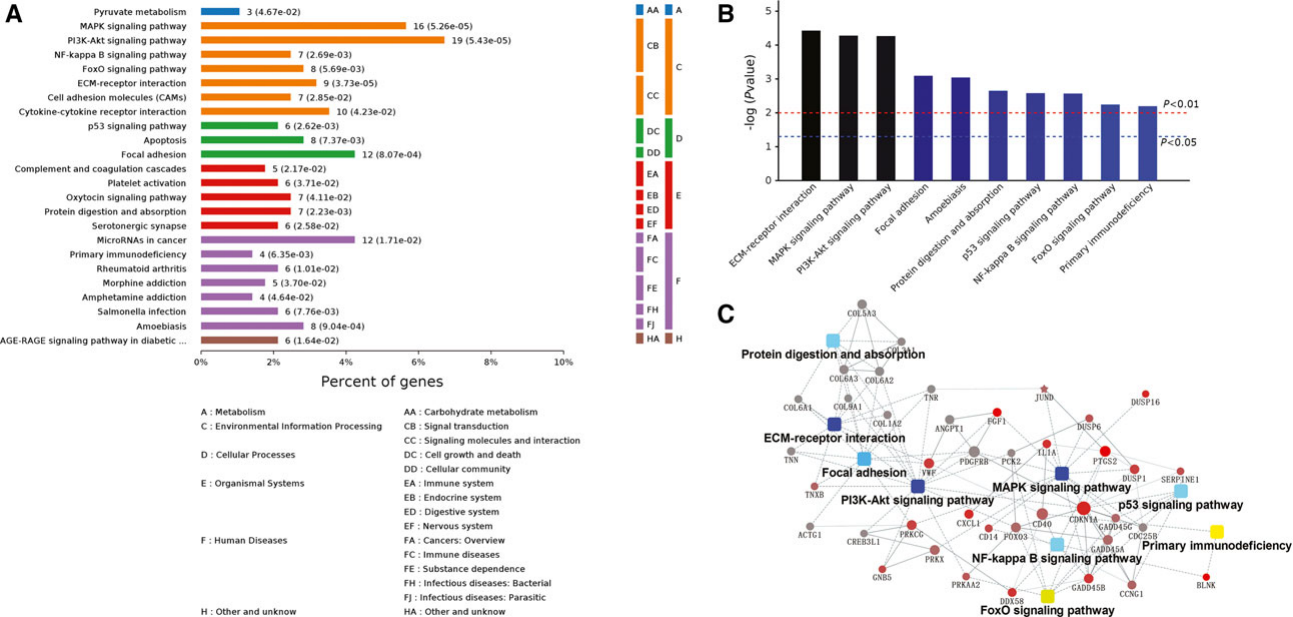
KEGG pathway analyses of RNA‐seq results. (A) The main KEGG pathways that the 385 changed genes lye in. (B) Ranking of top 10 pathways by *P* values. (C) Network analysis of the main signaling pathways that changed in the 30 p.p.m. FR L‐929 cells.

We also analyzed the signaling pathways that the 385 genes may lie in. Kyoto encyclopedia of genes and genomes (KEGG) pathway analyses showed that, of the six major functional areas, three of them were involved, which are ‘environmental information processing’, ‘organismal systems’, and ‘Human diseases’ (Fig. 6A). The top three pathways were ‘ECM‐receptor interaction’, ‘mitogen‐activated protein kinase (MAPK) signaling pathway’, and ‘phosphoinositide 3‐kinase (PI3K)‐Akt signaling pathway’. Some well‐studied pathways, such as ‘P53 signaling pathway’, ‘nuclear factor (NF)‐kappa B signaling pathway’, ‘forkhead box O (FoxO) signaling pathway’, and ‘Focal adhesion’, were also highly involved (Fig. 6B). Simulation of the complex signaling networks that may be involved in the fluoride resistance of L‐929 cells are shown in Fig. 6C.

### Validation of the gene expression change in the 30 p.p.m. FR L‐929 cells by real‐time RT‐PCR

**Figure 7:**
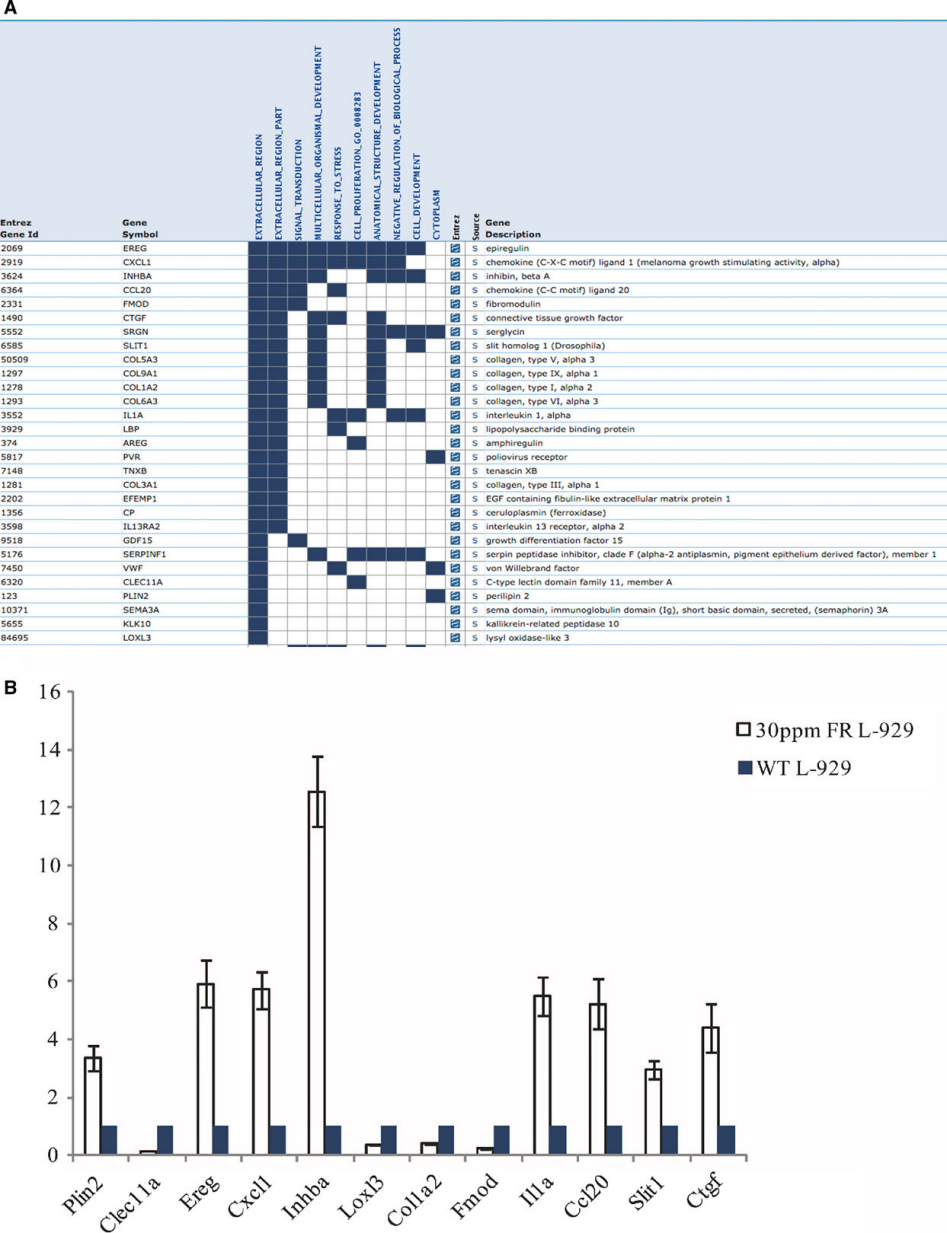
Validation of the key genes that were up‐ or down‐regulated in the 30 p.p.m. FR L‐929 cells. (A) List of the key genes that appear most frequently in the gene ontology analyses. (B) Validation of expression levels of the genes list in (A) by quantitative PCR. Data were represented as mean ± SD, *P < 0.05.

To validate the gene expression changes in the 30 p.p.m. FR L‐929 cells from the RNA‐seq data, we further performed real‐time RT‐PCR experiments on a list of genes that participated in multiple biological process and pathways concluded from our gene ontology and KEGG pathway studies (Fig. 7A). These genes could play very important roles in determining the fluoride resistance capabilities the L‐929 cells developed. As shown in Fig. 7B, expression changes in genes *Plin2*,* Clec11a*,* Ereg*,* Cxcl1*,* Inhba*,* Loxl3*,* Col1a2*,* Fmod*,* Il1a*,* Ccl20*,* Slit1*, and *Ctgf* were consistent with the expression fold change data from RNA‐seq studies. These data indicated that our RNA‐seq studies of the global gene expression changes in the 30 p.p.m. FR L‐929 cells were reliable.

## Discussion

Toxic effects of excessive fluoride on organisms including mammals have been well documented 7, 18. As a highly electronegative anion, fluoride is extremely active and tends to attract cations like calcium. Hence, the effect of fluoride on mineralized tissues such as bone and teeth leading to developmental alternations is of clinical significance; these mineralized tissues contain the highest amount of calcium and therefore attract fluoride that is incorporated to form calcium‐fluorapatite crystals. Excessive exposure to fluoride may interfere normal process of tissue mineralization; consequently, developmental alternations result in dental and skeletal fluorosis, two most common endemic fluorosis associated with excessive fluoride, which are harmful to individual health 8. There are more than 20 developed and developing nations in which fluorosis is endemic and the most severe problem occurs in Rift Valley countries in Africa, India, Sri Lanka and China 19, 20. Water source pollution or contamination with high levels of fluoride‐containing minerals is believed to be important reasons 15. It was noticed that even when exposed to the same levels of fluoride in some of those areas, endemic fluorosis does not appear to develop in a proportion of individuals, which indicates the existence of possible strategies that can protect individuals from fluoride toxicity. However, so far, little is known regarding the mechanisms responsible for such resistance or tolerance to fluoride toxicity in such individuals 9.

Recently, some new clues emerged with the discovery of the fluoride riboswitch (*crcB* RNA motif) that binds to fluoride and regulates the expression of genes in response to this anion in a wide variety of bacteria and archaea 16, 21. Riboswitches are metabolite or ion‐sensing, conservatively structured RNA motifs typically located in the noncoding regions of certain mRNAs. They control the expression of adjoining protein‐coding regions through several different mechanisms including transcription termination, translation blocking, and alternative splicing 22, 23, 24, 25. Fluoride riboswitches increase expression of downstream genes when fluoride levels are elevated, and the genes are proposed to help mitigate the toxic effects of very high levels of fluoride. Many genes are presumed to be regulated by these fluoride riboswitches. To date, two of the most common encode proteins that have been proposed to function by removing fluoride from the cell are identified, CrcB proteins and a fluoride‐specific subtype of chloride channels referred to as ClC^F^ 16, 21. ClC^F^ proteins have been shown to function as fluoride‐specific F^−^/H^+^ antiporters 14. Fluoride riboswitches are found in many organisms within the domains fungus, bacteria and archaea, indicating that many of these organisms sometimes encounter elevated levels of fluoride. However, no riboswitches have yet been identified in mammals which make the riboswitches more likely to be mechanisms specific to fungus, bacteria, and archaea but so far not to mammals.

Developing mammalian cells that can adapt to high level of fluoride is believed to be one of the best ways *in vitro* to investigate the mechanisms by which mammals can resist fluoride toxicity. Previous studies demonstrated that fluoride is cytotoxic and causes cell death when it reaches approximate 1 mm (19 p.p.m.) in culture media 26, 27, 28, 29, 30 depending upon pH 31 and cell types 32, 33, 34, 35. The results of the present study were in accord with previous reports. Only slight to moderate growth reduction was observed on WT L‐929 cells with 20 p.p.m. fluoride (1.052 mm) and could recover to normal within 2 weeks, while 30 p.p.m. (1.579 mm) and higher concentrations of fluoride caused more pronounced inhibition of cell growth. Fluoride above 60 p.p.m. (3.158 mm) killed almost all the cells within 1 day. Early studies in 1970s reported that by stepwise increase in fluoride concentration, cells adapted to grow at fluoride concentrations that would kill them 11. Several types of cells (e.g., L cells, LS cells, Hela cells, and CHO cells) that retained their vitality in lethal concentrations of fluoride were developed by slowly increasing the concentration of fluoride 10, 11, 30, 36, 37. However, due to limitations in research methodology, no further research was performed at molecular level to investigate mechanisms associated with the observation of fluoride resistance of cells.

In the present study, FR strains of L‐929 cells were successfully induced *in vitro* by sequential exposure of WT L‐929 cells to gradient ascending fluoride concentration in culture media. By using high‐throughput RNA‐sequencing technology, we, for the first time, systemically analyzed the global gene expression changes after fluoride adaptation of mammalian cells. We identified 385 genes that exhibited at least twofold changes in 30 p.p.m. FR mouse L‐929 cells compared with WT ones. Of the 385 genes, 265 genes were up‐regulated and 120 genes down‐regulated. Gene ontology analyses for these 385 genes showed that, response to stress, multiorganismal and system development, extracellular region of the cells, as well as protein and receptor binding are the main biological processes and molecular functions the FR L‐929 cells changed to adapt to the fluoride toxin. These data suggested that the high concentration of fluoride is firstly a strong stress for the mammalian cells to respond. The cells also tend to form a unity to adapt to the toxic stress and then stimulate downstream signaling pathways to up‐regulate the inner system of expelling the fluoride toxin. KEGG pathway analyses indicated that these downstream pathways that may play key roles in helping expelling fluoride out of cells are ‘ECM‐receptor interaction’, ‘MAPK signaling pathway’, ‘PI3K‐Akt signaling pathway’, ‘P53 signaling pathway’, ‘NF‐kappa B signaling pathway’, ‘FoxO signaling pathway’, and ‘Focal adhesion’ etc. These results further indicated that, even as simple as the addition of one toxin as an anion of fluoride, the cells respond to it in a very complex way to systemically change the functional units expressed within cells for adaptation or expelling the fluoride toxin.

Although we did not identify the single key gene/protein that determine fluoride resistance of mammalian cells in the current study, our approach using next‐generation RNA‐seq in FR mammalian cells should facilitate future studies in dissection of molecular pathways and the development of more appropriate FR mammalian cell model systems. We believe our data can be used as an important tool for novel genes and gene feature discovery in FR mammalian cells in later more detailed studies.

## Conclusion

In summary, under the limitation of this study, we developed FR strains of mouse L‐929 cells and systemically analyzed the global gene expression changes after fluoride adaptation of these mammalian cells. To our knowledge, our study showed, for the first time, that main biological processes and functions changed within the induced FR strains of L‐929 cells were associated with extracellular region and matrix, response to stress, receptor binding, and signal transductions etc., which indicate that high‐dose fluoride not only exerted stress on L‐929 cells but also induced functional pathways that helped them adapt to or expel the fluoride.

## Author contributions

BL and YL conceived and designed the project; SR, WZ, LW, and JW performed the experiments; NS, YL, and BL analyzed data; SR and NS wrote the paper; BL and NS made manuscript revisions. All authors read and approved the final manuscript.

## Supporting information


**Table S1** Transporters/Channels among the 385 changed genes. [Correction added after online publication on 21 June 2017: Table S1 revised].

## References

[1] Stauber T , Weinert S and Jentsch TJ (2012) Cell biology and physiology of CLC chloride channels and transporters. Compr Physiol 2, 1701–1744. 23723021 10.1002/cphy.c110038

[2] Jentsch TJ (2008) CLC chloride channels and transporters: from genes to protein structure, pathology and physiology. Crit Rev Biochem Mol Biol 43, 3–36. 18307107 10.1080/10409230701829110

[3] Zimmermann MB , Jooste PL and Pandav CS (2008) Iodine‐deficiency disorders. Lancet 372, 1251–1262. 18676011 10.1016/S0140-6736(08)61005-3

[4] ten Cate JM (1999) Current concepts on the theories of the mechanism of action of fluoride. Acta Odontol Scand 57, 325–329. 10777135 10.1080/000163599428562

[5] Hamilton IR (1990) Biochemical effects of fluoride on oral bacteria. J Dent Res 69 Spec No, 660–667; discussion 682–3. 2179327 10.1177/00220345900690S128

[6] Li S and Breaker RR (2012) Fluoride enhances the activity of fungicides that destabilize cell membranes. Bioorg Med Chem Lett 22, 3317–3322. 22460034 10.1016/j.bmcl.2012.03.006 PMC4498161

[7] Barbier O , Arreola‐Mendoza L and Del Razo LM (2010) Molecular mechanisms of fluoride toxicity. Chem Biol Interact 188, 319–333. 20650267 10.1016/j.cbi.2010.07.011

[8] Jagtap S , Yenkie MK , Labhsetwar N and Rayalu S (2012) Fluoride in drinking water and defluoridation of water. Chem Rev 112, 2454–2466. 22303811 10.1021/cr2002855

[9] Hardwick K , Barmes D and Richardson LM (2000) International collaborative research on fluoride. J Dent Res 79, 893–904. 10831090 10.1177/00220345000790040301

[10] Quissell DO and Suttie JW (1972) Development of a fluoride‐resistant strain of L cells: membrane and metabolic characteristics. Am J Physiol 223, 596–603. 5066349 10.1152/ajplegacy.1972.223.3.596

[11] Holland RI and Hongslo JK (1978) Fluoride, fluoride resistance and glycolysis in cultured cells. Acta Pharmacol Toxicol 43, 240–245. 10.1111/j.1600-0773.1978.tb02260.x 707137

[12] Fahlke C (2001) Ion permeation and selectivity in ClC‐type chloride channels. Am J Physiol Renal Physiol 280, F748–F757. 11292616 10.1152/ajprenal.2001.280.5.F748

[13] Miller C (2006) ClC chloride channels viewed through a transporter lens. Nature 440, 484–489. 16554809 10.1038/nature04713

[14] Stockbridge RB , Lim HH , Otten R , Williams C , Shane T , Weinberg Z and Miller C (2012) Fluoride resistance and transport by riboswitch‐controlled CLC antiporters. Proc Natl Acad Sci USA 109, 15289–15294. 22949689 10.1073/pnas.1210896109 PMC3458365

[15] Li S , Smith KD , Davis JH , Gordon PB , Breaker RR and Strobel SA (2013) Eukaryotic resistance to fluoride toxicity mediated by a widespread family of fluoride export proteins. Proc Natl Acad Sci USA 110, 19018–19023. 24173035 10.1073/pnas.1310439110 PMC3839697

[16] Baker JL , Sudarsan N , Weinberg Z , Roth A , Stockbridge RB and Breaker RR (2012) Widespread genetic switches and toxicity resistance proteins for fluoride. Science 335, 233–235. 22194412 10.1126/science.1215063 PMC4140402

[17] Stockbridge RB , Robertson JL , Kolmakova‐Partensky L and Miller C (2013) A family of fluoride‐specific ion channels with dual‐topology architecture. Elife 2, e01084. 23991286 10.7554/eLife.01084 PMC3755343

[18] Perumal E , Paul V , Govindarajan V and Panneerselvam L (2013) A brief review on experimental fluorosis. Toxicol Lett 223, 236–251. 24050947 10.1016/j.toxlet.2013.09.005

[19] Ayoob S and Gupta AK (2006) Fluoride in drinking water: a review on the status and stress effects. Crit Rev Environ Sci Technol 36, 433–487.

[20] Fawell JK and World Health Organization (2006) Fluoride in Drinking‐Water. IWA Pub, London, UK.

[21] Ren A , Rajashankar KR and Patel DJ (2012) Fluoride ion encapsulation by Mg2+ ions and phosphates in a fluoride riboswitch. Nature 486, 85–89. 22678284 10.1038/nature11152 PMC3744881

[22] Breaker RR (2009) Riboswitches: from ancient gene‐control systems to modern drug targets. Future Microbiol 4, 771–773. 19722830 10.2217/fmb.09.46 PMC5340290

[23] Roth A and Breaker RR (2009) The structural and functional diversity of metabolite‐binding riboswitches. Annu Rev Biochem 78, 305–334. 19298181 10.1146/annurev.biochem.78.070507.135656 PMC5325118

[24] Breaker RR (2011) Prospects for riboswitch discovery and analysis. Mol Cell 43, 867–879. 21925376 10.1016/j.molcel.2011.08.024 PMC4140403

[25] Breaker RR (2012) Riboswitches and the RNA world. Cold Spring Harb Perspect Biol 4, 1–13. 10.1101/cshperspect.a003566 PMC3281570 21106649

[26] Albright JA (1964) Inhibitory levels of fluoride on mammalian cells. Nature 203, 976. 10.1038/203976a0 14203515

[27] Albright JA (1966) Effect of fluoride on mammalian cells: partial reversal by pyruvate. J Oral Ther Pharmacol 2, 436–439. 5957280

[28] Carlson JR and Suttie JW (1967) Effects of sodium fluoride on HeLa cells. I. Growth sensitivity and adaption. Exp Cell Res 45, 415–422. 6021929 10.1016/0014-4827(67)90190-5

[29] Mankovitz R , Kisilevsky R and Florian M (1978) Chinese hamster cell lines resistant to the cytotoxic action of fluoride. Can J Genet Cytol 20, 71–84. 657004 10.1139/g78-009

[30] Hongslo JK , Holland RI and Jonsen J (1974) Effect of sodium fluoride on LS cells. J Dent Res 53, 410–413. 4521902 10.1177/00220345740530023801

[31] Helgeland K and Leirskar J (1976) pH and the cytotoxicity of fluoride in an animal cell culture system. Scand J Dent Res 84, 37–45. 2967 10.1111/j.1600-0722.1976.tb00459.x

[32] Armstrong WD , Blomquist CH , Singer L , Pollock ME and McLaren LC (1965) Sodium fluoride and cell growth. BMJ 1, 486–488. 14238676 10.1136/bmj.1.5433.486 PMC2165893

[33] Armstrong WD , Pollock ME and Singer L (1965) Sodium fluoride and cell growth. BMJ 1, 1435. 14289735 10.1136/bmj.1.5447.1435 PMC2166322

[34] Le Coultre‐Mulder GW , Veldhuizen C , Bouman J and Wise ME (1969) Influence of the fluorine ion on the growth in vitro of human amnion cells, T‐(kidney) cells, and HeLa cells. Acta Physiol Pharmacol Neerl 15, 1–19. 5772505

[35] Holland RI (1980) Cytotoxicity of fluoride. Acta Odontol Scand 38, 69–79. 6445672 10.3109/00016358009003481

[36] Holland RI and Hongslo JK (1978) Cellular resistance to fluoride. Cell Biol Int Rep 2, 551–559. 719772 10.1016/0309-1651(78)90063-2

[37] Carlson JR and Suttie JW (1967) Effects of sodium fluoride on HeLa cells. II. Metabolic alterations associated with growth inhibition. Exp Cell Res 45, 423–432. 6021930 10.1016/0014-4827(67)90191-7

