# Supplementary material for: Fluoride resistance capacity in mammalian cells involves complex global gene expression changes
Source: FEBS Open Bio. 2017 Jun 5;7(7):968–80. doi: 10.1002/2211-5463.12236 (PMC5494298; doi:10.1002/2211-5463.12236)
Supplement: Supplementary file 1 — Table S1 Transporters/Channels among the 385 changed genes. [Correction added after online publication on 21 June 2017: Table S1 revised]. [file FEB4-7-968-s001.docx]

**Table S1**, 385 changed genes.

| **Gene ID** | **log2 Fold Change** |
| --- | --- |
| Col6a1 | -2.9 |
| Col6a2 | -2.8 |
| Sema3c | 3.7 |
| Ptgs2 | 4.5 |
| Tnfrsf23 | 3.6 |
| Lcn2 | 5.9 |
| Pdk4 | 3.8 |
| H2-K1 | 1.8 |
| Efemp1 | -2.3 |
| Bgn | -2.2 |
| Cdkn1a | 2.6 |
| Gadd45b | 2.4 |
| H2-Q4 | 2.3 |
| Plin2 | 1.8 |
| Thbd | 2.0 |
| B2m | 1.7 |
| RP23-6I17.1 | 3.7 |
| Rn7sk | -4.7 |
| Cyr61 | 2.1 |
| Serpine1 | 1.9 |
| Gm11131 | 2.4 |
| Ccbe1 | 3.1 |
| Tnfrsf22 | 2.4 |
| C3 | 5.2 |
| Hist1h1c | 2.5 |
| Col5a3 | -1.9 |
| Kitl | 2.0 |
| Psmb8 | 2.4 |
| Clec11a | -2.7 |
| Col6a3 | -1.8 |
| Rnase4 | 4.2 |
| Ier3 | 2.6 |
| Col3a1 | -1.6 |
| Gda | 3.7 |
| Ereg | 2.6 |
| Havcr2 | 2.9 |
| Serpinf1 | -1.6 |
| Srgn | 1.9 |
| Efnb2 | 2.0 |
| Rarres2 | -1.8 |
| H2-T23 | 3.2 |
| Cadm4 | -1.8 |
| Cxcl1 | 2.5 |
| Ces1g | -4.2 |
| H2-D1 | 1.6 |
| Tgm2 | 1.9 |
| Cldn15 | -1.9 |
| Cdsn | -1.8 |
| Dusp6 | 1.8 |
| Dusp1 | 2.2 |
| Maff | 1.9 |
| Areg | 4.9 |
| Hmgn2 | -1.6 |
| Zfos1 | 1.6 |
| Gm20496 | 2.1 |
| Gdf15 | 1.8 |
| Pla1a | 3.2 |
| Gdpd5 | 3.3 |
| Phlda1 | 1.8 |
| 2410006H16Rik | 1.5 |
| Pdpn | 1.7 |
| Slpi | 2.3 |
| Akap12 | -2.1 |
| Lmo7 | 3.0 |
| Inhba | 3.7 |
| Serpinb9e | 3.2 |
| Gm28592 | 1.9 |
| Csn3 | 3.4 |
| Gsta4 | -1.8 |
| Actg1 | -1.4 |
| AU018091 | 3.1 |
| Trp53inp1 | 3.2 |
| 2810417H13Rik | -1.6 |
| Pvr | 1.6 |
| Lrrc61 | -1.6 |
| Hivep2 | 1.8 |
| Pcdh19 | -2.0 |
| Loxl3 | -1.6 |
| Gas1 | -1.7 |
| Tmem97 | -1.7 |
| Dusp16 | 2.6 |
| Slc27a6 | 3.3 |
| Sgms2 | 1.7 |
| Hist1h2bc | 2.5 |
| Cobl | 4.4 |
| Plin4 | -1.4 |
| Col1a2 | -1.3 |
| Gm2115 | -1.9 |
| Cited2 | 1.5 |
| Tnfrsf26 | 1.8 |
| Cbr2 | -2.0 |
| Acat1 | -1.5 |
| Sema3a | -1.8 |
| Gstm7 | -2.6 |
| Sdcbp2 | 2.4 |
| Snhg12 | 1.6 |
| Snhg1 | 1.4 |
| Nhs | -3.6 |
| Il13ra2 | 3.9 |
| Cyp4v3 | 2.6 |
| Gchfr | -1.8 |
| Angpt1 | -2.0 |
| Sdf2l1 | -1.6 |
| Snord43 | 3.6 |
| Sgk1 | 1.6 |
| Aldh3a1 | -2.2 |
| Gm20412 | 2.0 |
| Ppic | -1.4 |
| Tap2 | 1.5 |
| Fosb | 2.6 |
| Tnr | -2.6 |
| Pdgfrb | -1.5 |
| Ces1f | -3.9 |
| Sparcl1 | -2.4 |
| Psmb9 | 1.8 |
| Aig1 | 1.7 |
| Fzd4 | -1.6 |
| Col20a1 | 2.5 |
| Ndufa4l2 | -1.8 |
| Trim30a | 2.5 |
| Col9a1 | -3.5 |
| Cd80 | 2.7 |
| Ptpn22 | 2.8 |
| Vnn1 | 2.6 |
| Usp17la | 1.8 |
| Cth | -1.9 |
| Sh2d3c | -2.7 |
| Car9 | -1.7 |
| Fgf1 | 3.0 |
| H2-T10 | 3.2 |
| Ptprt | 2.9 |
| Grn | 1.3 |
| S100a1 | -1.4 |
| Irgm1 | 2.0 |
| Irgm2 | 3.5 |
| Arhgef28 | 2.3 |
| Ppp1r15a | 1.4 |
| Selp | 2.3 |
| Ifih1 | 3.3 |
| Cpe | 1.4 |
| Myo15 | 3.2 |
| Rem1 | -2.2 |
| Calcoco1 | 1.7 |
| Tecta | 3.1 |
| Dab2 | -1.5 |
| Oaf | -1.5 |
| H2-M3 | 2.0 |
| Apol9b | 2.6 |
| Srgap3 | 1.5 |
| Nos2 | 3.4 |
| F5 | 3.4 |
| Cd14 | 2.0 |
| Oas3 | 2.9 |
| Serpinb9f | 2.4 |
| Cisd3 | -1.4 |
| Oasl1 | 2.5 |
| H2-Q1 | 1.7 |
| Cgnl1 | 2.2 |
| Cp | -1.4 |
| Stom | 1.3 |
| Bpifc | 3.2 |
| Ccng1 | 1.3 |
| Gm10800 | 2.7 |
| Acot2 | 1.4 |
| Igfbp6 | -1.2 |
| Xaf1 | 3.0 |
| Tapbp | 1.3 |
| Tnxb | 1.4 |
| Sprr2h | 3.3 |
| Ddx58 | 2.4 |
| Fam171b | 2.1 |
| Cd40 | 1.5 |
| Creb3l1 | -1.4 |
| 9-Sep | -1.3 |
| Gcnt2 | 1.5 |
| Kng2 | 2.8 |
| G0s2 | -2.3 |
| Tmem154 | 2.3 |
| Apol7a | 2.1 |
| Gm15958 | 2.8 |
| 1810011O10Rik | 3.1 |
| Parp14 | 2.4 |
| Plb1 | 2.5 |
| Enpp2 | 2.9 |
| Map1a | -1.5 |
| Olfml3 | -1.5 |
| Alpl | 3.1 |
| Blnk | 3.0 |
| Gm15821 | 1.5 |
| Hist2h2bb | 2.6 |
| Igtp | 3.0 |
| Trerf1 | -1.9 |
| Cyp3a13 | 3.1 |
| Tmcc3 | 1.8 |
| Pik3c2a | 1.3 |
| Sncg | -1.3 |
| Plscr2 | 2.4 |
| Lif | 1.7 |
| Gper1 | -2.1 |
| Gm9574 | 3.0 |
| Prr32 | 2.2 |
| Trp53inp2 | 1.6 |
| Sp100 | 2.3 |
| Abca1 | 1.9 |
| Serinc1 | 1.2 |
| Trp53cor1 | 2.0 |
| S100a13 | -1.4 |
| Glipr1 | 1.4 |
| Ctsk | -1.4 |
| Pde1b | 2.6 |
| H2-T22 | 1.6 |
| Atp8b4 | -1.8 |
| Hopx | -2.2 |
| Gnb5 | 2.0 |
| mt-Rnr1 | -1.8 |
| Slc35f1 | 2.7 |
| Ifi44 | 2.8 |
| Cd274 | 2.2 |
| Oas1b | 2.5 |
| Dusp9 | -1.5 |
| Usp18 | 2.7 |
| Gm11175 | 1.4 |
| Sox9 | 1.6 |
| Lpcat4 | -1.7 |
| Ifit2 | 1.5 |
| Prkcg | 2.2 |
| Gbp2 | 2.7 |
| Snord104 | -2.1 |
| Pcx | -1.5 |
| Enpp5 | 1.4 |
| Gm19684 | 2.8 |
| Mogat1 | 2.6 |
| Atg9b | 2.4 |
| 9930111J21Rik2 | 1.8 |
| Gm8995 | 2.5 |
| Jund | 1.3 |
| Celsr3 | 2.4 |
| Rhbdd1 | 1.3 |
| Vwa5a | 1.4 |
| Psmd8 | 1.2 |
| Tcp11l2 | 2.0 |
| Pde4b | 2.2 |
| Chit1 | 2.7 |
| Zbtb8b | -2.2 |
| Sec16b | -1.6 |
| Gbp2b | 2.7 |
| Loxl4 | 1.7 |
| Oas1a | 1.9 |
| Klhl30 | 2.2 |
| Cox6a2 | -2.1 |
| Trim7 | 1.9 |
| Pim1 | 1.3 |
| Gm11974 | 1.4 |
| Spaca6 | -1.5 |
| Hcar2 | 2.7 |
| Arsj | -2.0 |
| Btg2 | 1.8 |
| Ankle1 | -1.7 |
| Olfml2b | -2.3 |
| Snap25 | -2.6 |
| Emp3 | -1.6 |
| Prkar2b | -1.2 |
| Lgals2 | -2.3 |
| Fmod | -2.0 |
| Eva1b | -1.6 |
| Camsap3 | -1.5 |
| Ablim1 | 1.6 |
| Il1a | 2.5 |
| Slc6a12 | 2.6 |
| Slc16a8 | 2.0 |
| Asah2 | 1.7 |
| Gm44250 | 1.4 |
| Fam189a1 | 2.5 |
| Lce1g | 2.5 |
| Ackr3 | 1.7 |
| Pvt1 | 1.3 |
| Nhlrc3 | 1.3 |
| Tap1 | 1.2 |
| Notch4 | 2.1 |
| Serpinb6b | 1.4 |
| Perm1 | 1.7 |
| Dynlt3 | 1.3 |
| Lrrn4cl | 1.6 |
| Gm43003 | 1.8 |
| Foxo3 | 1.2 |
| Kalrn | 2.0 |
| Aim1l | 2.3 |
| Snora28 | 2.2 |
| Tigit | 2.0 |
| Gjb4 | 1.5 |
| Rgs10 | -1.8 |
| Tnfrsf9 | 1.6 |
| Ptrf | -1.1 |
| Cd9 | 2.3 |
| Aifm2 | 1.4 |
| RP23-121N17.3 | -1.8 |
| Pxdn | 2.4 |
| Pid1 | -1.5 |
| Lyz2 | -2.1 |
| Herc6 | 2.1 |
| Vwf | 2.3 |
| Acacb | 1.5 |
| Gm26885 | 1.5 |
| Adra2a | 2.4 |
| Sprr2g | 2.4 |
| Ccl20 | 2.4 |
| Rab26os | -1.8 |
| Prkaa2 | 1.7 |
| B230303O12Rik | 1.9 |
| Gpm6a | -2.3 |
| Notch3 | -1.8 |
| Napb | 1.6 |
| Pck2 | -1.2 |
| Lmnb1 | -1.2 |
| Hist1h2al | 2.1 |
| Gm43566 | -2.0 |
| Arsb | 1.3 |
| Klk10 | -1.5 |
| Map2 | -2.3 |
| Lrp1 | -1.1 |
| Eda2r | 2.1 |
| Try5 | 2.2 |
| Tspan32 | -1.5 |
| Pax5 | 1.5 |
| Trim56 | 1.2 |
| Platr3 | -1.9 |
| Avpi1 | -1.3 |
| Cdh23 | 1.9 |
| Gadd45a | 1.2 |
| Crebl2 | 1.5 |
| Gadd45g | 1.2 |
| Dhrs3 | 1.6 |
| Msln | 1.2 |
| Scara3 | -1.6 |
| Naa10 | -1.7 |
| Samd9l | 1.5 |
| Cdc25b | -1.2 |
| Adamts6 | 2.2 |
| Gm17300 | -1.2 |
| Prickle1 | 1.5 |
| Skil | 1.2 |
| Serpinb9 | 1.3 |
| Cnksr1 | 1.9 |
| Prkx | 1.2 |
| C920025E04Rik | 1.6 |
| Hr | 1.6 |
| Tnn | -1.7 |
| Plcxd2 | 1.4 |
| Nhsl2 | -2.1 |
| RP24-365N15.2 | 2.2 |
| Slc52a2 | -1.3 |
| Adam33 | -1.8 |
| Cd200r4 | 2.2 |
| Cacna2d2 | -2.1 |
| Gamt | -1.7 |
| BC023719 | 2.2 |
| Ifi35 | 1.5 |
| Tns1 | -1.1 |
| Gm20655 | 1.8 |
| Dnah10 | 2.1 |
| Gm7785 | 2.1 |
| Obscn | 2.0 |
| Crip1 | -1.5 |
| Cystm1 | 1.4 |
| Vgf | -1.4 |
| Slit1 | 1.6 |
| Krt17 | 1.9 |
| Trp63 | 1.6 |
| Ypel3 | 1.5 |
| Lct | 2.0 |
| Mgmt | 1.6 |
| Ctso | 2.0 |
| Btn1a1 | 2.1 |
| Rpph1 | -1.8 |
| Sema4f | 2.1 |
| Ctgf | 2.1 |
| Adamts10 | -1.4 |
| Ptgs1 | 1.3 |
| Elavl3 | 1.8 |
| Vmn2r13 | 2.1 |
| Lbp | 1.5 |
| Lrrn3 | -1.9 |
| RP24-144C5.1 | 2.1 |
| B130024G19Rik | -1.7 |
| Chst1 | -1.2 |
